# miR-211 suppresses epithelial ovarian cancer proliferation and cell-cycle progression by targeting Cyclin D1 and CDK6

**DOI:** 10.1186/s12943-015-0322-4

**Published:** 2015-03-11

**Authors:** Bairong Xia, Shanshan Yang, Tianbo Liu, Ge Lou

**Affiliations:** Department of Gynecology, the Affiliated Tumor Hospital, Harbin Medical University, 150 Haping Rd, Nangang, Harbin, 150020 Heilongjiang, China

**Keywords:** CDK6, Cyclin D1, Epithelial ovarian cancer, miR-211

## Abstract

**Background:**

Epithelial ovarian cancer (EOC) is a significant cause of morbidity and mortality. MicroRNAs play important roles in cancer development and progression. The microRNA miR-211 is localized on intron 6 of the *Trpm1* gene at 15q13-q14, a locus that is frequently lost in neoplasms. Its function and loss-of-function have been described in normal and cancer cells and tissues. miR-211 is known to be dysregulated in ovarian cancer: however, its function and the downstream effect of its loss-of-function in ovarian cancer have not been described before.

**Methods:**

We analyzed miR-211 expression in clinical samples of primary EOC tissues compared to normal epithelial ovarian tissues and in the EOC cell lines: OVCAR3, Caov3, OVCA429, SKOV3 and A2780 compared to human ovarian surface epithelial cells. We then investigated the effect of miR-211 on EOC cell proliferation and apoptosis by counting cell numbers, MTT, colony formation, cell cycle, and PI/Annexin V staining assays. A luciferase reporter system was developed to assess miR-211 regulation of the predicted targets. Expression level of discovered targets and correlation with miR-211 expression were analyzed in EOC tissues. Finally, OVCAR3 stably expressing miR-211 or control cells were injected subcutaneously into mice to determine *in vivo* effect of miR-211 on tumorigenesis.

**Results:**

We found that the expression of miR-211 is downregulated in EOC tissues and cell lines compared to normal epithelial ovarian tissue and human ovarian surface epithelial cells, respectively. miR-211 was found to arrest cells in the G0/G1-phase, inhibit proliferation and induce apoptosis. Cyclin D1 and CDK6 were found to be direct targets of miR-211, and when overexpressed in miR-211-expressing EOC cells, could restore proliferative ability. Finally, *in vitro* investigation confirmed that miR-211 is a tumor suppressor that controls Cyclin D1 and CDK6 expression.

**Conclusions:**

Our results demonstrate that miR-211 is a tumor suppressor that controls expression of Cyclin D1 and CDK6, and that its downregulation results in overexpression of Cyclin D1 and CDK6 which increases proliferation ability of EOC cells to proliferate compared to normal cells.

**Electronic supplementary material:**

The online version of this article (doi:10.1186/s12943-015-0322-4) contains supplementary material, which is available to authorized users.

## Background

Ovarian cancer (OC) has a high mortality rate and low 5-year survival rate due to lack of early, safe and non-invasive detection methods. This malignancy also develops chemoresistance during recurrence after initial chemotherapy [1-4]. Therefore, new therapies, clinical biomarkers and treatment targets are in demand.

MicroRNAs (miRNAs) regulate gene expression at the post-transcriptional level [5-7] and miRNA dysregulation is frequently associated with cancer progression, including OC [8-12]. The microRNA miR-211 is localized on intron 6 of the *Trpm1* gene at 15q13-q14, a locus that is frequently lost in neoplasms [13-16]. MiR-211 functions and the effect of loss-of-function have been described in normal and cancer cells and tissues. Using mouse embryonic fibroblasts, Chitnis et al. [17] found that miR-211 is a pro-survival molecule that is expressed in a PERK (aka EIF2AK3, Eukaryotic translation initiation factor 2-alpha kinase) -dependent manner and regulates the expression of *chop/gadd153* by mediating temporal accumulation of the pro-apoptotic transcription factor *chop*. PERK is important to survival of tumor and normal cells in response to stress [18-22] and Chitnis et al. [17] suggested that miR-211 negatively regulates chop accumulation, allowing cells to re-establish homeostasis before having to commit to apoptosis.

In clinical melanoma samples, Mazar et al. [8] found that miR-211 targets KCNMA1, is downregulated in melanoma and that its expression is microphthalma-associated transcription factor dependent. This transcription factor is important for melanocyte growth, maturation, apoptosis and pigmentation [23]. Bell et al. found that miR-211 contributes to melanoma adhesion by targeting the AMP-activated protein kinase-related kinase NUAKI and that inhibition of miR-211 resulted in increased NUAK1 expression and reduced adhesion [24]. In glioblastoma multiform, miR-211 was found to be downregulated with an inverse correlation of miR-211 expression and matrix metalloproteinase-9 expression [25]. The authors suggested that rescuing miR-211 expression could have therapeutic applications. Conversely, others reported that in oral carcinoma, miR-211 is upregulated, contributes to progression of oral carcinoma and correlates with poor prognosis in oral carcinoma [26].

The present study investigated the regulatory role and implications of aberrant expression of miR-211 in epithelial OC (EOC). We report that miR-211 is downregulated in EOC, inhibits proliferation and induces apoptosis in EOC cells *in vitro* and that overexpression of miR-211 inhibits growth of EOC xenograft tumors *in vivo* by repressing Cyclin D1 and CDK6 expression.

## Results

### miR-211 is downregulated in EOC tissues and cell lines

**Figure 1:**
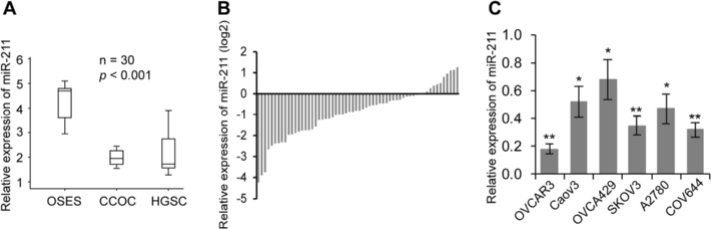
**miR-211 is downregulated in epithelial ovarian cancer (EOC) tissues and cell lines. A**. Expression of miR-211 was lower in clear cell ovarian carcinomas (CCOC, n = 9) and high-grade serous ovarian carcinomas (HGSC, n = 12) compared to ovarian surface epithelial cells (OSEC, n = 9) (GSE47841, p < 0.001). **B**. Levels of miR-211 were lower in 60 EOC tissues compared to 20 normal epithelial tissues samples. **p < 0.01. Relative levels of miRNA expression normalized to U6 snRNA was determined by setting the miRNA expression levels of normal epithelial tissue samples to 1. **C**. miR-211 expression was downregulated in six EOC cell lines (OVCAR3, Caov3, OVCA429, SKOV3, A2780 and COV644) compared to human ovarian surface epithelial (HOSE) cells. Relative levels of miRNA expression normalized to U6 snRNA was determined by setting the miRNA expression levels of HOSE cells to 1. *p < 0.05, **p < 0.01. Data are presented as mean ± SEM of three independent experiments.

Searching the literature, we found that miR-211 is downregulated in OC tissues [9]. We further used a public data base to investigate miR-211 expression in EOC tissues and found that the of miR-211 expression was significantly lower in clear-cell OC (CCOC, n = 9) and high-grade serous ovarian carcinomas (HGSC, n = 12) than in ovarian surface epithelial cells (OSES, n = 9) (Figure 1A, GSE47841, *p* < 0.001) [27]. Next, we explored this finding in clinical samples by comparing miR-211 expression in normal epithelial ovarian tissues with primary EOC tissues. Consistent with the mentioned literature, miR-211 expression was significantly lower in EOC tissues than in normal epithelial ovarian tissues (Figure 1B, *p* < 0.01). We extended our investigations to six EOC cell lines (OVCAR3, Caov3, OVCA429, SKOV3, A2780 and COV644) and found that their miR-211 expression levels were significantly lower than those of human ovarian surface epithelial (HOSE) cells (Figure 1C). These findings suggest that downregulation of miR-221 may affect EOC development.

### miR-211 inhibits proliferation and induces apoptosis of EOC cells

**Figure 2:**
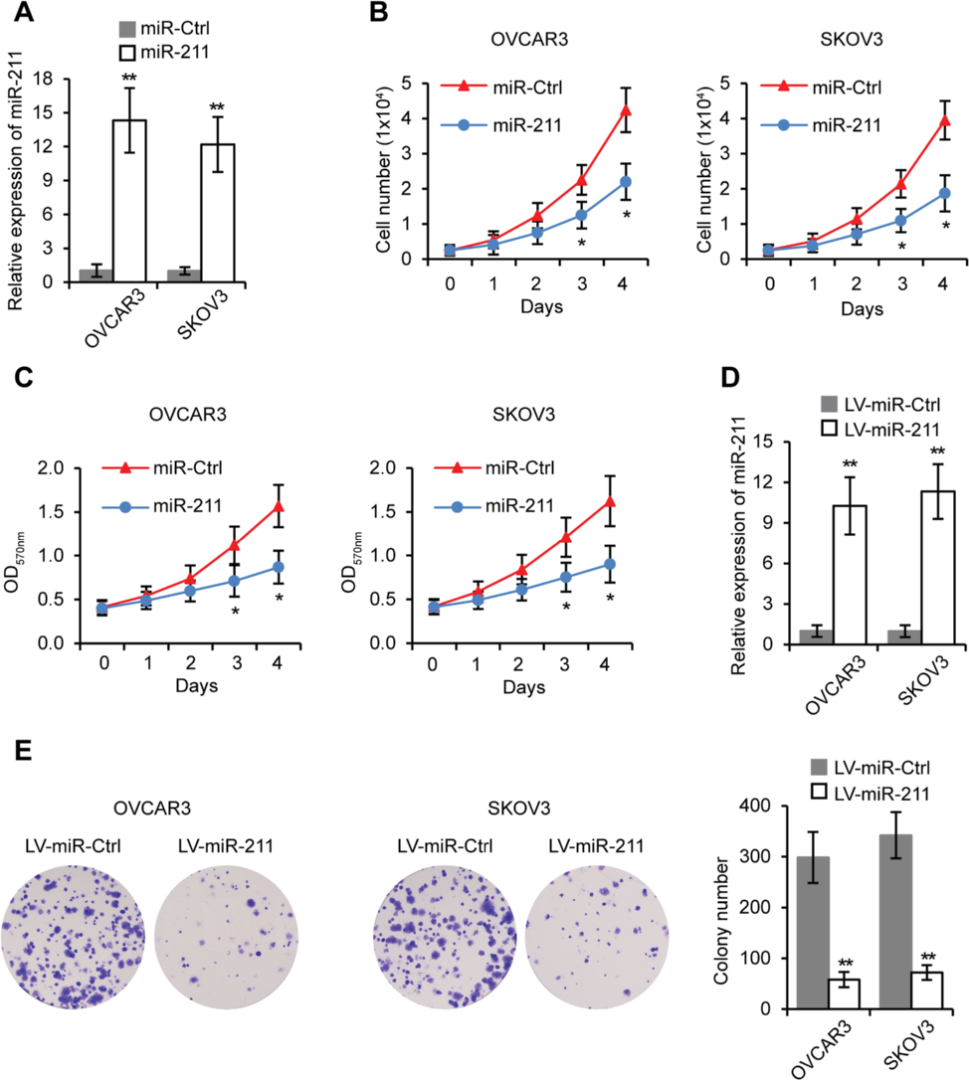
**miR-211 inhibits EOC cell proliferation. A**. miR-211 levels in OVCAR3 and SKOV3 cells transfected with miR-211 or miR-Ctrl. **B**. OVCAR3 and SKOV3 cells were transfected with miR-211 or miR-Ctrl for 48 hours, then seeded in 24-well plates (0.25 × 10^4^ cells/well). Viable cell numbers were counted at indicated time points. **C**. MTT assay of OVCAR3 and SKOV3 cells transfected with miR-211 or miR-Ctrl. **D**. Expression of miR-211 in LV-miR-211 or LV-miR-Ctrl transfected OVCAR3 and SKOV3 cells. **E**. Colony formation assay in OVCAR3 and SKOV3 cells stably expressing miR-211 compared to control cells. *p < 0.05, **p < 0.01 compared to miR-Ctrl or LV-miR-Ctrl transfected cells. Data are presented as mean ± SEM of three independent experiments.

To investigate the function of miR-211 in EOC tumorigenesis, we transfected miR-211 or miR-Ctrl in OVCAR3 and SKOV3 cell lines and determined their miR-211 levels 48 hours after transfection. Results showed increased miR-211 levels in both miR-211 transfected cell lines compared to miR-Ctrl transfected cells (Figure 2A). We then investigated the effect of miR-211 on OVCAR3 and SKOV3 cell proliferation. As shown in Figure 2B, miR-211 significantly inhibited EOC proliferation. MTT assay further confirmed that miR-211 had a negative effect on EOC cell proliferation (Figure 2C). To examine the effect of miR-211 on long-term EOC cell proliferation, we performed colony formation assays. We first constructed a miR-211 lentiviral vector (LV-miR-211), then infected OVCAR3 and SKOV3 cells with LV-miR-211 to establish stably expressing miR-211 cells (Figure 2D). LV-miR-211 and LV-miR-Ctrl cells were subjected to colony formation assay for two weeks. As expected, miR-211 significantly reduced colony numbers to 20% in both EOC cell lines (Figure 2E).

**Figure 3:**
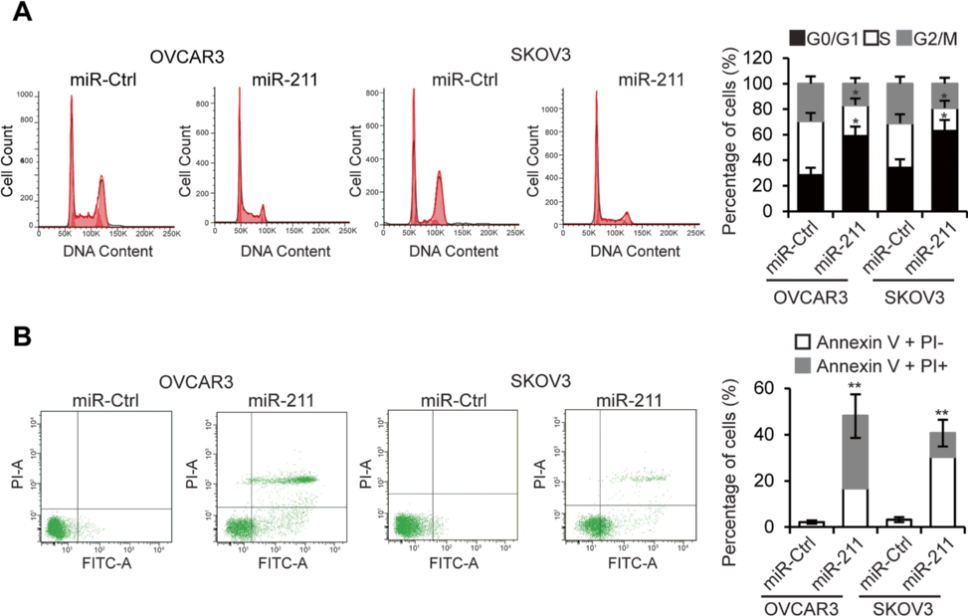
**miR-211 arrests EOC cell cycle and induces apoptosis. A**. Cell cycle assay of OVCAR3 and SKOV3 cells transfected with miR-211 or miR-Ctrl. **B**. Apoptosis analysis of OVCAR3 and SKOV3 cells transfected with miR-Ctrl or miR-211. *p < 0.05, **p < 0.01 compared to miR-Ctrl transfected cells. Data are presented as mean ± SEM of three independent experiments.

Next, we performed cell cycle analysis and noted that miR-211 transfection significantly arrested significantly more cells in the G0/G1 phase (Figure 3A) than miR-211-Ctrl transfection. To investigate whether miR-211 affects EOC cell apoptosis, OVCAR3 and SKOV3 cells were transfected with miR-211 and apoptosis assessed 48 hours later. As shown in Figure 3B, miR-211 transfected cells had a higher incidence of apoptosis than miR-211-Ctrl transfected cells. These results suggest that miR-211 inhibits EOC cell proliferation and induces apoptosis.

### Cyclin D1 and CDK6 are direct target of miR-211

**Figure 4:**
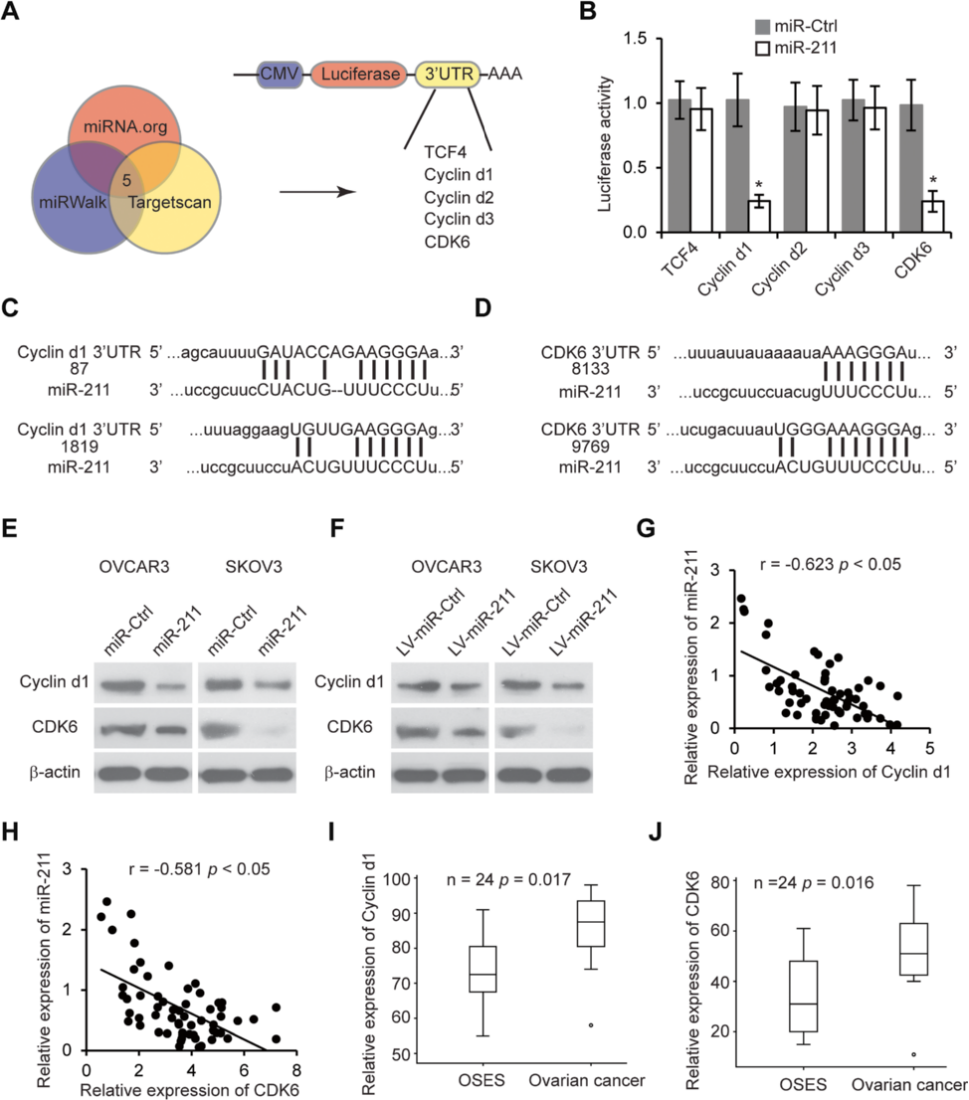
**Cyclin D1 and CDK6 are miR-211 targets. A**. Schematic representation showing the five predicted miR-211 targets (TCF4, Cyclin D1, Cyclin D2, Cyclin D3 and CDK6) screened using three algorithms (miRNA.org, miRWalk and Targetscan). **B**. OVCAR3 cells were co-transfected with TCF4, CDK6, Cyclin D1, Cyclin D2 or Cyclin D3 3′UTR pGL plasmid with miR-211. Forty-eight hours later, luciferase assay was performed. *p < 0.05 compared to miR-Ctrl transfected cells. Data are presented as mean ± SEM of three independent experiments. **C**-**D**. Sequence of miR-211 with the putative binding sites of Cyclin D1 **(C)** and CDK6 (D) 3′ UTR. **E**. Western blot of Cyclin D1 and CDK6 levels in OVCAR3 and SKOV3 cells transfected with miR-211 or miR-Ctrl. **F**. Western blot of Cyclin D1 and CDK6 levels in OVCAR3 and SKOV3 cells transfected with LV-miR-211 or LV-miR-Ctrl. **G**-**H**. The mRNA expression levels of Cyclin D1 **(G)** or CDK6 **(H)** correlated inversely with miR-211. **I**-**J**. Cyclin D1 **(I)** and CDK6 **(J)** expression in ovarian tissues (GDS3592/Cyclin D1, n = 24, *p* = 0.017; GDS3592/CDK6, n = 24, *p* = 0.016).

We used microRNA.org, Targetscan and miRWalk databases to predict potential miR-211 targets. Hundreds of potential targets were found, but we selected transcription factor 4 (TCF4), Cyclin D1, Cyclin D2, Cyclin D3 and Cyclin dependent kinase 6 (CDK6) for further analysis since these genes have previously been reported to affect EOC cell proliferation [28-31] (Figure 4A). We inserted TCF4, CDK6, Cyclin D1, Cyclin D2 and Cyclin D3 3′UTR into luciferase reporter vectors and co-transfected with miR-211 expression plasmid into OVCAR3 and SKOV3 cells (Figure 4A). Forty-eight hours after transfection, only cells transfected with CDK6 and Cyclin D1 3′UTR plasmids had lower luciferase activity than their controls. Luciferase activity in both of these cell lines was 70% less than that of the miR-Ctrl groups (Figure 4B). Next, we compared the 3′UTR sequence of Cyclin D1 and CDK6 with miR-211. Both genes have two potential complementary miR-211 binding sites (Figure 4C, D). We next constructed Cyclin D1 and CDK6 3′UTR mutant plasmid (Additional file 1: Figure S1A-B), cotransfected with miR-211 and then performed luciferase assay. The data revealed that miR-211 targets two sites in Cyclin D1 and CDK6 (Additional file 1: Figure S1C-D). To confirm that Cyclin D1 and CDK6 are specifically targeted by miR-211, OVCAR3 and SKOV3 cells transfected with miR-211 or infected with LV-miR-211 were subjected to western blot analysis. We found that the protein levels of Cyclin D1 and CDK6 were lower in miR-211 and LV-miR-211 transfected cells compared to control (Figure 4E, F). The mRNA level of Cyclin D1 and CDK6 and the levels of miR-211 were measured in 60 EOC tissues using qRT-PCR to analyze their correlation. Spearman correlation analysis revealed a reverse correlation between miR-211 expression and Cyclin D1 or miR-211 and CDK6 expression (Figure 4G, H). The high expression of Cyclin D1 and CDK6 in OEC tissues compared to normal tissue was consistent with results from the public database (Figure 4I, J) [32]. Taken together, these results indicate that miR-211 can repress the expression of Cyclin D1 and CDK6 in EOC by directly targeting the 3′UTR of Cyclin D1 and CDK6 mRNA.

### miR-211 affects ovarian cell proliferation and apoptosis through suppression of Cyclin D1 and CDK6

**Figure 5:**
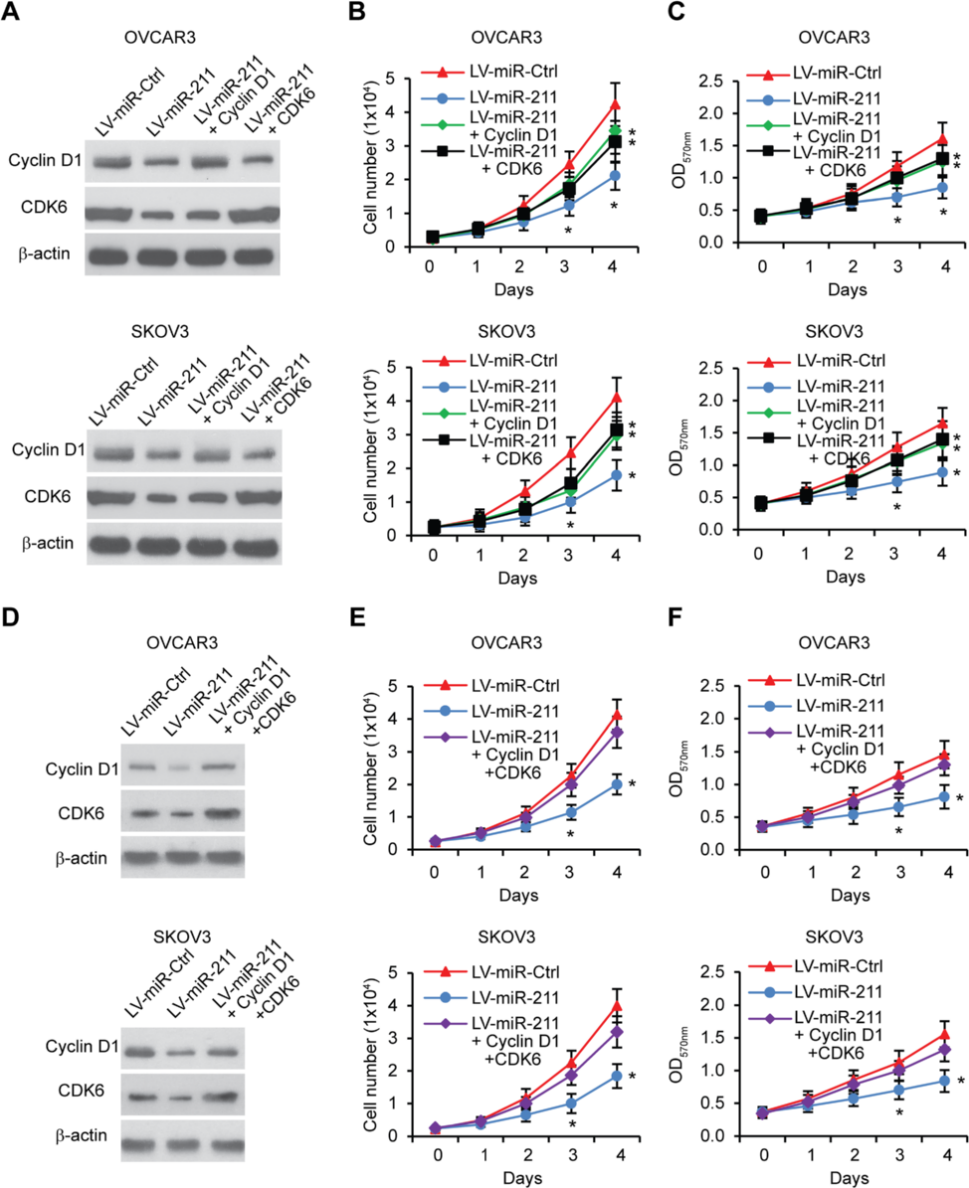
**miR-211 affects EOC cell proliferation through suppression of Cyclin D1 and CDK6. A**. Western blot of Cyclin D1 and CDK6 levels in OVCAR3 and SKOV3 cells transfected with LV-miR-Ctrl, LV-miR-211, LV-miR-211 + Cyclin D1 or LV-miR-211 + CDK6 plasmids. **B**. Cell counting of OVCAR3 and SKOV3 cells transfected with LV-miR-Ctrl, LV-miR-211, LV-miR-211 + Cyclin D1 or LV-miR-211 + CDK6 plasmids. **C**. MTT assay of OVCAR3 and SKOV3 cells transfected with LV-miR-Ctrl, LV-miR-211, LV-miR-211 + Cyclin D1 or LV-miR-211 + CDK6 plasmids. **D**. Western blot of Cyclin D1 and CDK6 levels in OVCAR3 and SKOV3 cells transfected with LV-miR-Ctrl, LV-miR-211 or LV-miR-211 + Cyclin D1 + CDK6 plasmids. **E**. Cell counting of OVCAR3 and SKOV3 cells transfected with LV-miR-Ctrl, LV-miR-211 or LV-miR-211 + Cyclin D1 + CDK6 plasmids. **F**. MTT assay in OVCAR3 and SKOV3 cells transfected with LV-miR-Ctrl, LV-miR-211 or LV-miR-211 + Cyclin D1 + CDK6 plasmids. *p < 0.05 compared to LV-miR-Ctrl transfected cells. Data are presented as mean ± SEM of three independent experiments.

**Figure 6:**
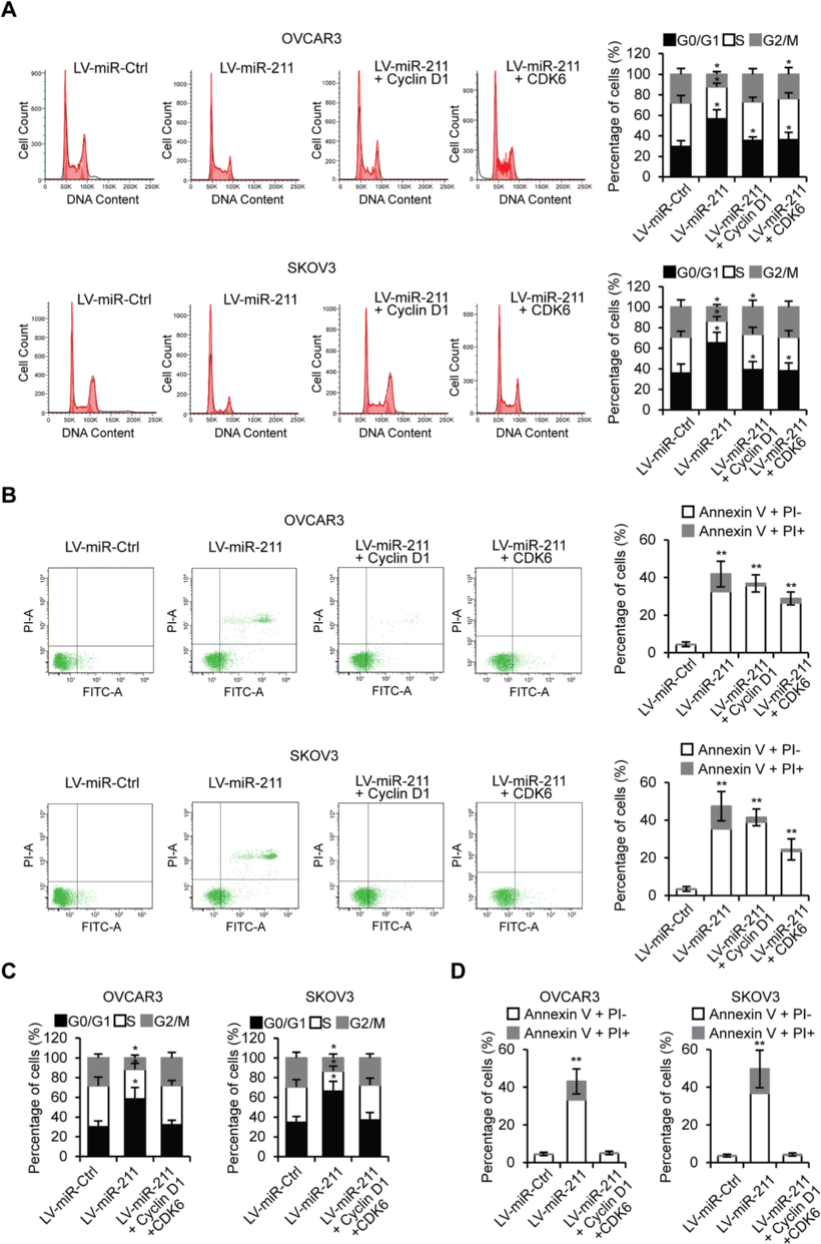
**miR-211 regulates EOC cell cycle and cell apoptosis via repression of Cyclin D1 and CDK6. A**. Cell cycle assay of OVCAR3 and SKOV3 cells tranfected with LV-miR-Ctrl, LV-miR-211, LV-miR-211 + Cyclin D1 or LV-miR-211 + CDK6 plasmids. **B**. Apoptosis analysis of OVCAR3 and SKOV3 cells transfected with LV-miR-Ctrl, LV-miR-211, LV-miR-211 + Cyclin D1 or LV-miR-211 + CDK6 plasmids. **C**. Cell cycle assay of OVCAR3 and SKOV3 cells tranfected with LV-miR-Ctrl, LV-miR-211 or LV-miR-211 + Cyclin D1 + CDK6 plasmids. **D**. Apoptosis analysis of OVCAR3 and SKOV3 cells transfected with LV-miR-Ctrl, LV-miR-211 or LV-miR-211 + Cyclin D1 + CDK6 plasmids. *p < 0.05, **p < 0.01 compared to LV-miR-Ctrl transfected cells. Data are presented as mean ± SEM of three independent experiments.

Our previous data suggests that miR-211 inhibits EOC cell proliferation and that Cyclin D1 and CDK6 are direct targets of miR-211. To demonstrate that miR-211 regulates cell proliferation through Cyclin D1 and CDK6, we tested whether Cyclin D1 and CDK6 could rescue the impaired proliferative phenotype in miR-211 overexpressing OVCAR3 and SKOV3 cells. Western blot was performed to examine Cyclin D1 and CDK6 levels in cells transfected with LV-miR-Ctrl, LV-miR-211, LV-miR-211 + Cyclin D1 and LV-miR-211 + CDK6 (Figure 5A). Cell counting and MTT assay were performed to analyze cell proliferation. The results indicate that miR-211 inhibited proliferation, while Cyclin D1 or CDK6 partly, and Cyclin D1 + CDK6 almost completely restored cell proliferation (Figure 5B-F). Furthermore, we performed cell cycle assays and found that Cyclin D1 or CDK6 partly rescued the cell proliferation that had been inhibited by miR-211 (Figure 6A). MiR-211 overexpression led to more cell apoptosis, while Cyclin D1 and CDK6 each significantly reduced apoptosis (Figure 6B). When we overexpressed Cyclin D1 and CDK6 in miR-211-overexpressing cells, miR-211-induced cell cycle arrest and apoptosis were completely abrogated (Figure 6C and D). These results together demonstrate that miR-211 affects EOC cell proliferation, at least in part, through suppression of Cyclin D1 and CDK6.

### miR-211 reduces EOC tumorigenesis in vivo

**Figure 7:**
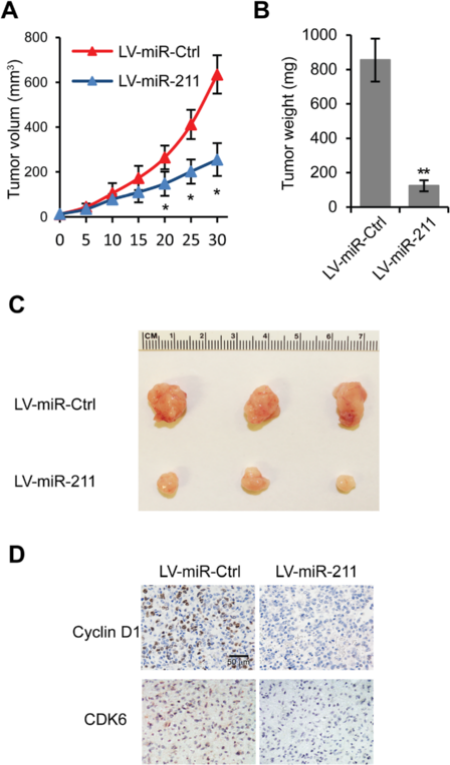
**miR-211 reduces EOC tumorigenesis** ***in vivo.*** **A**. Nude mice were implanted with OVCAR3 cells stably expressing miR-Ctrl or miR-211. Tumor volume was measured every five days. **B**. Tumor weight determined at day 25. *p < 0.05, **p < 0.01 compared to LV-miR-Ctrl transfected cells. Data are presented as mean ± SEM. **C**. Representative images of formed tumors. **D**. Immunohistochemistry of Cyclin D1 and CDK6 staining in LV-miR-Ctrl and LV-miR-211 groups.

We performed *in vivo* experiments to confirm our *in vitro* results that suggested that miR-211 inhibited EOC cell proliferation by targeting Cyclin D1 and CDK6. Sixteen mice were randomly divided into two groups. OVCAR3 cells stably expressing miR-211 or control cells were injected subcutaneously into mice in each group. We found that tumor growth was slower in the LV-miR-211 group compared to the LV-miR-Ctrl group (Figure 7A). The tumor weights and sizes were smaller in LV-miR-211 group compared to LV-miR-Ctrl group (Figure 7B, C). Finally, these tumor tissues were assessed with immunohistochemistry. We observed that Cyclin D1 and CDK6 staining in LV-miR-211 group was weaker than in the control group (Figure 7D). These *in vivo* results further indicated that miR-211 inhibits EOC growth and reduces Cyclin D1 and CDK6 expression.

## Discussion

MiRNAs are undoubtedly pivotal to tumorigenesis and understanding their functions may help provide new cancer therapies [33-36]. In the present study, we performed a database search for miR-211 expression in human ovarian cancer tissues compared to healthy control tissue, and found that miR-211 was significantly downregulated in clear-cell and high-grade serous carcinomas. This was further confirmed in clinical primary EOC samples and in EOC cell lines.

We further investigated the significance of miR-211 expression in EOC *in vitro* and found that miR-211 significantly modulated EOC cell proliferation and colony formation. Cell cycle analysis showed that miR-211 arrested cells in the G0/G1 phase, resulting in apoptosis. Using bioinformatics, we identified several miR-211 targets and confirmed with luciferase assay that miR-211 directly binds to sequences in Cyclin D1 and CDK6 mRNA, repressing their translation into protein. Further *in vitro* investigations showed that miR-211 affected EOC cell proliferation and apoptosis through suppressing the expression of Cyclin D1 and CDK6.

We confirmed our *in vitro* observations *in vivo* with a mouse tumor model. As expected, we found that Cyclin D1 and CDK6 were downregulated *in vivo* by miR-211 and that EOC tumor growth was reduced significantly by miR-211 overexpression.

Dysregulated expression of CDK6 and Cyclin D1 has been reported in several cancers, including head and neck squamous cell carcinoma, non-small cell lung carcinoma, endometrial cancer, melanoma, pancreatic cancer, breast cancer, colorectal cancer, mantle cell lymphoma, multiple myeloma, prostate cancer, endometrial cancer and oesophageal cancer (Cyclin D1, [37]), and glioblastoma, myxofibrosarcoma, lymphoid malignancies and Ewing’s sarcoma cell line (CDK6, [38-42]).

We did not investigate the effect of dysregulated CDK6 and Cyclin D1 on downstream gene expression; however, both have been ascribed several functions. Cyclin D1 controls CDK6 activity and is known to affect angiogenesis, respond to growth factor stimulation and stimulates G1 progression. Overexpression of Cyclin D1 (and other Cyclins) was found to shorten the G1-phase of the cell cycle in various cell types [43-45] and inhibiting Cyclin D1 in human fibroblasts was found to inhibit progression through G1 [45,46], which is consistent with our observations in EOC*.* Also, D-type Cyclin overexpression can surmount the G1 growth arrest caused by retinoblastoma tumor suppressor protein (Rb) in Saos-2 osteosarcoma cells [47,48]. Although Cyclin D1 interacts with CDK6 to exert many of its functions, it also performs CDK6-independent functions such as: transcriptional regulation leading to cell growth, tissue-specific differentiation and cell cycle progression, as well as chromatin modifications and interaction with nuclear hormone receptors which both lead to differentiation and androgen-receptor-dependent cell cycle progression (reviewed by [37]).

CDK6 is a kinase catalytic subunit of a protein kinase complex that is involved in G1 progression and G1/S transition. CDK6 activity first occurs in mid G1-phase, is controlled by D-type Cyclins (i.e. Cyclin D1) and INK4 family members, and regulates Rb activity by phosphorylation [49]. Phosphorylation of Rb leads to the release of E2fs, which then activate transcription of genes required for S-phase entry [50]. Very recently, Handschick et al. [51] reported that CDK6 is a co-factor of NF-κB that interacts physically with the NF-κB subunit p65 and is found at promoters of NF-κB target genes. Thus, dysregulated CDK6 and Cyclin D1 expression is significant as it is likely to affect expression of S-phase entry proteins, and the cytokine and chemokine expression profile of EOC, contributing to oncogenesis and tumorigenesis.

CDK6 overexpression increases cell proliferation and reduces DNA repair activity by accelerating G1/S -phase progression. In glioma, CDK6 knockdown was found to increase sensitivity to chemotherapy [52]. On this basis, it is possible that miR-211-mediated inhibition of CDK6 expression in EOC could be a useful epigenetic therapeutic approach, although further experiments would be required to determine this.

In summary, we found that miR-211 negatively regulates CDK6 and Cyclin D1 activity and that miR-211 is downregulated in EOC, leading to aberrant expression of CDK6 and Cyclin D1, which results in loss of cell cycle control. Cyclin D1 and CDK6 appear to be key players in EOC tumorigenesis, and our discovery of correlated expression of miR-211 and CDK6/Cyclin D1 provides new insight that presents tentative methods for diagnosis, prognosis and therapy for EOC, and a rational for further investigation into the potential use of miR-211 for diagnosis and therapy.

## Materials and methods

### Human samples

This study was approved by the Medical Ethics Committee of Harbin Medical University Cancer Hospital and all patients provided informed consent. Tissues were collected from patients who underwent surgery at the Department of Obstetrics and Gynecology of Harbin Medical University Cancer Hospital between 2012 and 2013, including 60 epithelial EOC tissues and 20 normal epithelial ovarian tissue sections. Patients with previous radiation therapy, chemotherapy, or immunotherapy, were excluded from the study. The histopathological diagnostics was performed according to the World Health Organization criteria. All fresh specimens were stored at −80°C for further use. Patients’ characteristics including presenting age, clinical stage, pathological stage and tumor size are available in Table 1.

**Table 1:** **Clinical characteristics of 60 EOC patients**

**Feature**	**Cases (%)**
Age (years)	
≤55	26 (43.3%)
>55	34 (56.7%)
Clinical stage	
I-II	25 (43.3%)
III-IV	35 (56.7%)
Pathological grade	
1-2	22 (36.7%)
3	38 (63.3%)
Tumor size (cm)	
≤1	28 (46.7%)
>1	32 (53.3%)

### Cancer cell lines and primary normal epithelial cells

The human EOC cell lines (OVCAR3, Caov3, OVCA429, SKOV3 and A2780) and normal Human Ovarian Surface Epithelial (HOSE) cells were acquired from the China Center for Type Culture Collection (CCTCC). The COV644 cell line was purchased from Sigma (St. Louis, MO). EOC cells were cultured in Dulbecco’s modified Eagle’s medium (DMEM; Gibco-BRL, Gaithersburg, MD) supplemented with 10% fetal bovine serum and antibiotics (Gibco). HOSE cells were cultured in medium containing 1:1 mixture of MCDB 105 and M199 medium (Sigma). All cells were incubated at 37°C in a humidified atmosphere containing 5% CO_2_.

### Quantitative real-time PCR (qRT-PCR)

Total RNA was extracted using Trizol reagent (Invitrogen, Carlsbad, CA). To quantitate miR-211 expression, total RNA was polyadenylated and reverse transcribed using the TaqMan MicroRNA Reverse Transcription Kit and TaqMan miRNA assay (Applied Biosystems, Foster City, CA), according to the manufacturer’s instructions. U6 small nuclear RNA was used as the internal control. qRT-PCR analyses for mRNA of Cyclin D1 and CDK6 were performed using QIAGEN OneStep RT-PCR kits (Qiagen, Valencia, CA). The mRNA level of β-actin was measured as an internal control. RT-PCR was performed in triplicates. Relative expression of the tested genes was calculated and normalized using the 2^−ΔΔCt^ method. Primers were as follows: Cyclin D1 forward, 5′ GAGACCATCCCCCTGACGGC 3′, reverse, 5′ TCTTCCTCCTCCTCGGCGGC 3′; CDK6 forward, 5′ CGAATGCGTGGCGGAGATC 3′, reverse, 5′ CCACTGAGGTTAGAGCCATC 3′; β-actin forward, 5′ TGACGGGGTCACCCACACTGTGCCCATCTA3′, reverse, 5′ CTAGAAGCATTTGCGGTGGACGATGGAGGG 3′.

### miRNA, lentivirus production, plasmid and transfection

Oligonucleotides including miR-211 miRNA, mimics and non-specific miRNA negative control (miR-Ctrl) [53] were synthesized and purified by GenePharma (Shanghai). All oligonucleotides were transfected into EOC cells at a final concentration of 50 nM using HiPerFect transfection reagent according to the product manual (Qiagen). The human miR-211 precursor sequences were cloned into the lentivirus based expression plasmid pSILK (Addgene, Cambridge, MA). Lentiviruses were packaged by transfecting HEK293T cells with the lentivirus vector pSLIK, packing plasmid psPAX2 and envelop plasmid pMD2.G (Addgene) in a 4:2:1 ratio using Lipofectamine 2000 (Invitrogen). After 48 hours, the medium containing the lentiviruses was collected. OVCAR3 cells were transduced with 1 × 10^6^ IFU/ml of lentiviruses in 8 μg/ml of polybrene (Sigma) for 16 hours*.* Forty-eight hours later, 150 μg/ml Hygromycin B was added to the medium and replenished every two days for four weeks to select the cells infected with the lentivirus. The full-length 3′UTR of Cyclin D1 and CDK6 gene containing the putative miR-211 biding sites was amplified by PCR and was inserted into the pGL3 vector subcloned with CMV promoter (Promega, Madison, WI). The coding sequences of Cyclin D1 and CDK6 were generated by PCR and cloned into pCDNA3.1(+) vector (Invitrogen) to generate pCDNA3.1-Cyclin D1 and pCDNA3.1-CDK6. Correct insertion of PCR-amplified sequences was confirmed by sequencing. The plasmid was transfected using Lipofectamine LTX according to the manufacturer’s instructions.

### Cell counting and 3-(4, 5-dimethylthiazolyl-2-yl)-2-5 diphenyl tetrazolium bromide (MTT) assay

The cell viability and proliferation were determined by cell counting and MTT assay (Promega). For cell counting, at 48 hours after transfection, 0.25 × 10^4^ cells were seeded into 24-well plates. Then cells were trypsinized and counted at 0, 1, 2, 3 and 4 days. For MTT assay, 2000 cells per well in a final volume of 100 μl were plated in 96-well plates 48 hours after transfection. Then at 0, 1, 2, 3 and 4 days, 25 μl of MTT stock solution was added to each well and incubated for 4 hours. The absorbance was measured at 570 nm. The assays were performed in triplicates.

### Colony formation assay

Forty-eight hours after infection with LV-miR-Ctrl or LV-miR-211, the EOC cells were seeded in 6-well plates (500 cells per well) and incubated for 2 weeks for the colony formation assay. The cells were then washed with PBS, fixed with 10% formalin, and stained with 0.5% crystal violet (Sigma). The assay was repeated in triplicates.

### Cell cycle assay

Forty-eight hours after transfection with miRNA mimics, EOC cells were seeded in 6-well plates. Two days later, the cells were collected and fixed in 70% ethanol, washed in PBS, re-suspended in 200 μl of PBS containing 0.5 mg/ml RNase, 0.05% Triton X-100 and 10 μg/ml propidium iodide (Sigma), incubated for 1 hour at 37°C in the dark, and analyzed immediately using a Flow Cytometer (BD Biosciences, San Jose, CA). The experiment was done in triplicates.

### Luciferase reporter assay

The cells were seeded in triplicate in 24-well plates one day before transfection for the luciferase assays. Plasmids inserted into the Renilla lucifearse vector (Promega) with Cyclin D1 or CDK6 3′UTR inserts were co-transfected with miR-Ctrl or miR-211 plasmids. Forty eight hours after transfection, the cells were harvested and lysed, and the luciferase activity assayed using the dual-luciferase assay kit (Promega). Normalized luciferase activity was reported as luciferase activity/Renilla luciferase activity. Three independent experiments were performed.

### Western blot

Total protein was extracted using RIPA buffer (50 mM Tris–HCl pH 7.4,150 mM NaCl, 1% NP-40, 1% sodium deoxycholic acid, 0.1% SDS, 1 mM phenylmethylsulfonyl fluoride, protease inhibitor cocktail; Santa Cruz, Santa Cruz, CA). The total extracts were separated using 10% SDS-polyacrylamide gels and electrophoretically transferred to polyvinylidene difluoride membranes (PVDF, Bio-Rad, Hercules, CA). The membranes were probed with a primary antibodies against human CDK6, Cyclin D1 and β-actin (Santa Cruz), followed by HRP-conjugated sencondary antibody (Santa Cruz). Bound antibodies were detected using the Supersignal West Pico ECL chemiluminescence kit (Thermo scientific, Rockford, IL).

### Animal studies

Animal studies were approved by the Institutional Animal Care and Use Committee of Harbin Medical University. Nude mice (5 weeks old) were randomly divided into two groups (*n* = 8 per group). A suspension of OVCAR3 cells (1 × 10^7^) stably expressing miR-211 or cells infected with miR-Ctrl were injected subcutaneously into the left flank of each group. Tumor volumes were measured every 5 days using a caliper. Thirty days after implantation, the mice were sacrificed and the subcutaneous tumors excised and weighed.

### Immunohistochemistry

Tumor samples were fixed in 4% formaldehyde, embedded in paraffin wax, and then cut into 5 μm sections. Samples were deparaffinized in clearite and rehydrated. After blocking endogenous peroxidase and performing antigen retrieval, tissue slides were blocked in goat serum for 30 min and incubated with antibodies against Cyclin D1 or CDK6 (1:100 dilution) overnight at 4°C, followed by biotinylated secondary antibody (Santa Cruz) for 30 min. Staining was performed in parallel using a Vectastain ABC kit (Vector Laboratories).

### Statistical analysis

The statistical analyses were performed using SPSS Windows version 19. Data is expressed as mean ± SEM of triplicate experiments. One-Way ANOVA was performed to determine significant differences between groups. Differences were considered significant when *p* < 0.05 (*) and highly significant when *p* < 0.01 (**).

## Additional file

Additional file 1:
**miR-211 targets multiple sites of Cyclin D1 and CDK6 3′UTR.** Sequence of miR-211 with the putative binding sites of Cyclin D1 3′UTR wild type and mutant A. CDK6 3′UTR wild type and mutant B-C. Luciferase assay in OVCAR3 cells co-transfected with miR-211 and Cyclin D1 3′UTR wild type or Cyclin D1 3′UTR mutant plasmid. D. Luciferase assay of OVCAR3 cells co-transfected with miR-211 and CDK6 3′UTR wild type or CDK6 3′UTR mutant plasmid. **p* < 0.05 compared to miR-Ctrl and 3′UTR wild type plasmid co-transfected cells. Data are presented as mean ± SEM of three independent experiments.

## Abbreviations

CCOC: Clear cell ovarian carcinomas
CDK6: Cyclin dependent kinase 6
EOC: Epithelial ovarian cancer
HGSC: High-grade serous ovarian carcinomas
HOSE: Human ovarian surface epithelial
OC: Ovarian cancer
OSES: Ovarian surface epithelial cells
TCF4: Transcription factor 4
