# Supplementary figures and images for: miR-211 suppresses epithelial ovarian cancer proliferation and cell-cycle progression by targeting Cyclin D1 and CDK6
Source: Mol Cancer. 2015 Mar 11;14:57. doi: 10.1186/s12943-015-0322-4 (PMC4359570; doi:10.1186/s12943-015-0322-4)

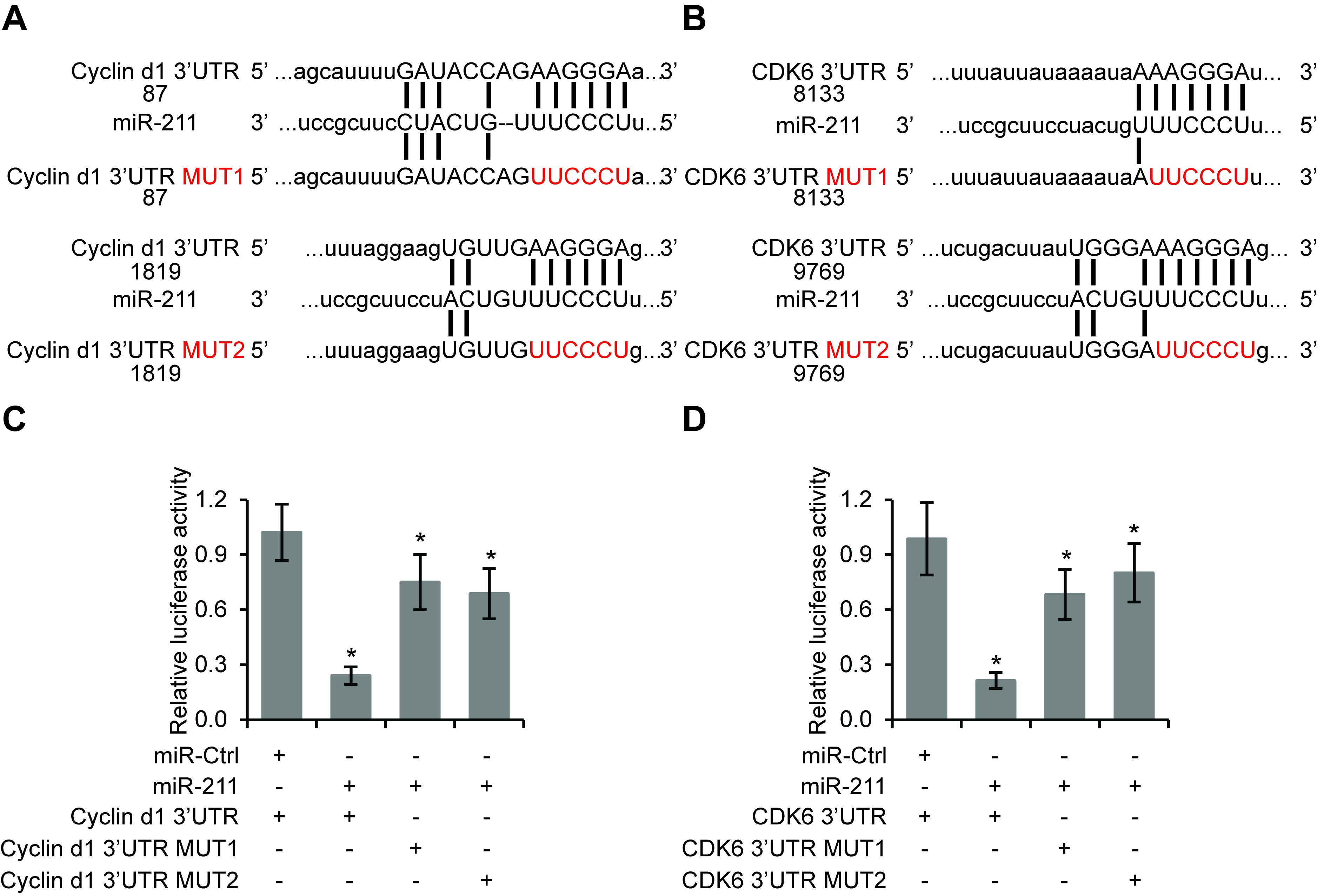

Supplement: Additional file 1: — miR-211 targets multiple sites of Cyclin D1 and CDK6 3′UTR. Sequence of miR-211 with the putative binding sites of Cyclin D1 3′UTR wild type and mutant A. CDK6 3′UTR wild type and mutant B-C. Luciferase assay in OVCAR3 cells co-transfected with miR-211 and Cyclin D1 3′UTR wild type or Cyclin D1 3′UTR mutant plasmid. D. Luciferase assay of OVCAR3 cells co-transfected with miR-211 and CDK6 3′UTR wild type or CDK6 3′UTR mutant plasmid. *p < 0.05 compared to miR-Ctrl and 3′UTR wild type plasmid co-transfected cells. Data are presented as mean ± SEM of three independent experiments. [file 12943_2015_322_MOESM1_ESM.tiff]
